# Involvement of Transcription Elongation Factor GreA in *Mycobacterium* Viability, Antibiotic Susceptibility, and Intracellular Fitness

**DOI:** 10.3389/fmicb.2020.00413

**Published:** 2020-03-23

**Authors:** Siyuan Feng, Yan Liu, Wanfei Liang, Mohamed Abd El-Gawad El-Sayed Ahmed, Zihan Zhao, Cong Shen, Adam P. Roberts, Lujie Liang, Liya Liao, Zhijuan Zhong, Zhaowang Guo, Yongqiang Yang, Xin Wen, Hongtao Chen, Guo-bao Tian

**Affiliations:** ^1^Department of Microbiology, Zhongshan School of Medicine, Sun Yat-sen University, Guangzhou, China; ^2^Key Laboratory of Tropical Diseases Control, Ministry of Education, Sun Yat-sen University, Guangzhou, China; ^3^Clinical Laboratory, Fifth Affiliated Hospital, Sun Yat-sen University, Zhuhai, China; ^4^Department of Microbiology and Immunology, Faculty of Pharmaceutical Sciences and Drug Manufacturing, Misr University for Science and Technology, Cairo, Egypt; ^5^Department of Tropical Disease Biology, Liverpool School of Tropical Medicine, Liverpool, United Kingdom; ^6^Centre for Drugs and Diagnostics, Liverpool School of Tropical Medicine, Liverpool, United Kingdom; ^7^School of Pharmaceutical Sciences (Shenzhen), Sun Yat-sen University, Guangzhou, China

**Keywords:** *greA*, *Mycobacterium tuberculosis*, *Mycobacterium smegmatis*, bacterial fitness, antibiotic susceptibility

## Abstract

There is growing evidence that GreA aids adaptation to stressful environments in various bacteria. However, the functions of GreA among mycobacteria remain obscure. Here, we report on cellular consequences following deletion of *greA* gene in *Mycobacterium* spp. The *greA* mutant strain (Δ*greA*) was generated in *Mycobacterium smegmatis*, *Mycobacterium tuberculosis* (MTB) H37Ra, and *M. tuberculosis* H37Rv. Deletion of *greA* results in growth retardation and poor survival in response to adverse stress, besides rendering *M. tuberculosis* more susceptible to vancomycin and rifampicin. By using RNA-seq, we observe that disrupting *greA* results in the differential regulation of 195 genes in *M. smegmatis* with 167 being negatively regulated. Among these, KEGG pathways significantly enriched for differentially regulated genes included tryptophan metabolism, starch and sucrose metabolism, and carotenoid biosynthesis, supporting a role of GreA in the metabolic regulation of mycobacteria. Moreover, like *Escherichia coli* GreA, *M. smegmatis* GreA exhibits a series of conservative features, and the anti-backtracking activity of C-terminal domain is indispensable for the expression of *glgX*, a gene was down-regulated in the RNA-seq data. Interestingly, the decrease in the expression of *glgX* by CRISPR interference, resulted in reduced growth. Finally, intracellular fitness significantly declines due to loss of *greA*. Our data indicates that GreA is an important factor for the survival and resistance establishment in *Mycobacterium* spp. This study provides new insight into GreA as a potential target in multi-drug resistant TB treatment.

## Introduction

*Mycobacterium tuberculosis* (MTB), the causative agent of tuberculosis (TB) has influenced human populations since ancient times. In 2018, *M. tuberculosis* caused seven million TB cases and was responsible for 1.5 million deaths worldwide (Harding, 2019). The administration of anti-TB drugs has led to the emergence of drug-resistant strains of MTB (Kumar et al., 2017) with approximately 5% of infections being caused by multidrug-resistant (MDR) strains globally (Wright et al., 2009). China has a high prevalence of drug-resistant TB and is the region with second largest number of MDR cases worldwide (Yang et al., 2017). Therefore, exploring the cause of MDR-TB will be pivotal within the implementation of effective public health strategies to reduce MDR-TB.

Transcription (the first step of gene expression) is fundamentally important to all life. Once RNA polymerase (RNAP) initiates the process of transcription, it is vital for the enzyme to carry out elongation and termination to ensure full-length RNA synthesis. Several transcription factors can bind to RNAP, modifying its properties by affecting transcription processivity and fidelity through modulating pausing, arrest, and termination (Tehranchi et al., 2010; Li et al., 2015). The transcript cleavage factor, GreA, interacts with the RNAP secondary channel and stimulates the intrinsic transcript cleavage activity of RNAP for the removal of the aberrant RNA 3′ ends. Therefore, polymerization activity can be restarted from the end of a cleaved RNA allowing transcription to resume (Komissarova and Kashlev, 1997).

Gre factors are widely distributed in prokaryotes. There is growing evidence that Gre factors participate in each step during the transcription process, including initiation, elongation, and fidelity (Yuzenkova et al., 2014; Traverse and Ochman, 2018). In addition, it has been reported that GreA is essential for the survival of bacteria under stress (Volker et al., 1994; Nogales et al., 2002). Moreover, GreA, which can facilitate RecBCD-mediated resection and inhibits RecA, plays an important role in impeding DNA break repair in *Escherichia coli* (Sivaramakrishnan et al., 2017), indicating a role for GreA in adapting to stressful environments.

The genome of *M. tuberculosis* contains a Gre-factor encoded by *rv1080c*. Recent *in vitro* experiments demonstrated that *Mycobacterium smegmatis* GreA exhibits chaperone-like activity (Joseph et al., 2019). It has been shown that *M. tuberculosis* GreA failed to rescue *E. coli* RNAP stalled elongation complexes (China et al., 2011). This suggested that the function of *M. tuberculosis* GreA is sufficiently different from the other well-studied bacterial GreA systems to warrant further investigation. Gumber et al. (2009) revealed that *greA* was found to be upregulated in anaerobic and acidic conditions in *Mycobacterium avium*. Moreover, further studies showed that *greA* was upregulated in *M. tuberculosis* following treatment with ofloxacin, moxifloxacin, and streptomycin (Sharma et al., 2010; Lata et al., 2015), indicating an important role for GreA in the environmental adaptation.

Here, we aimed to dissect the role of GreA and determine any influence on the fitness of mycobacteria. We revealed that GreA serves as a transcriptional factor that coordinates the expression of genes involved in metabolism in *M. smegmatis*. Our data suggests that GreA may be a novel target for adjunctive therapy.

## Materials and Methods

### Ethics Statement

All animal experiments were performed in accordance with the National Institutes of Health Guide for the Care and Use of Laboratory Animals, and the experimental procedures were approved by the Ethics Committee of Zhongshan School of Medicine on Laboratory Animal Care (reference number: 2016-159), Sun Yat-sen University.

### Animal Studies in C57BL/6 Mice

Female C57BL/6 mice were purchased from Animal Supply Center, Sun Yat-sen University. Mice were bred and maintained under specific pathogen-free conditions at the animal facility of the Sun Yat-sen University. Adult mice between 6–8 weeks of age were used, and infected mice were maintained under biosafety two conditions. Mice were infected with 1 × 10^7^ colony-forming units (CFUs) of H37Ra, Δ*greA* and *comp-*Δ*greA*, through the intraperitoneal injection. Four mice from each group were sacrificed after 14- and 21- days post-infection, and the lung and spleen homogenates (prepared by homogenizing aseptically removed organs in 1 ml of sterile saline) were plated on 7H10 agar supplemented with 10% OADC (BD Biosciences), in triplicates. CFUs of *M. tuberculosis* were counted after 3-4 weeks of incubation at 37°C.

### Bacterial Strains and Generation of *greA* Mutants

A mutant (Δ*greA*) strain of *M. tuberculosis* was constructed as described previously (Jain et al., 2014). For the deletion of *greA* in *M. tuberculosis* H37Rv, PCR was performed to synthesize fragments bearing the 1000 and 980 bps of flanking regions of endogenous *greA* of *M. tuberculosis*, resulting in a deletion of the gene (primer set Mtb *greA*-LL and Mtb *greA*-LR for the 5′ region and primer set Mtb *greA*-RL and Mtb *greA*-RR for the 3′ flanking region). Amplicons corresponding to upstream and downstream flanking regions were digested with *Van*91I and cloned into the *Van*91I digested p0004s plasmid that contains a hygromycin resistance cassette and the *sacB* gene to be able to select for sucrose sensitivity. This allelic exchange substrate was introduced into the *Pac*I site of phasmid phAE159 and electroporated into *M. smegmatis* mc2^1^ 155 to obtain high titers of phage phAE159. Subsequently, the *M. tuberculosis* wild-type strain was incubated with high titers of the corresponding phage to create *greA* knockouts. Colonies that had deleted endogenous *greA* were selected on hygromycin-containing plates and verified for sucrose sensitivity. Since the H37Ra genome is highly similar to that of H37Rv with respect to gene content, similar experiments have been performed for generation of H37Ra mutant strain. For unmarking of the deletion mutations generated by the helper plasmid pYUB870 (SacB, tnpR, and KanR). After transformation by electroporation with pYUB870 and plating onto medium containing kanamycin, 5 kanamycin-resistance colonies were obtained and screened by steaking on 7H10 agar alone and on 7H10 agar with 50 μg/ml hygromycin. A hygromycin-sensitive clone was grown in liquid medium in the absence of antibiotic selection. Further confirmation of *res-hyg-sacB-res* cassette deletion was achieved by PCR analysis and sequencing.

For the construction of *M. smegmatis* Δ*greA* strain, an in-frame deletion of the gene was enabled using Xer site-specific recombination as previously described (van Kessel and Hatfull, 2007). Briefly, two DNA fragments, approximately 500 bp, flanking *greA* of *M. smegmatis* were acquired through PCR and cloned at the borders of the hygromycin excisable cassette. The resulting DNA fragment was purified and electroporated into the competent cells of *M. smegmatis* harboring pJV53 plasmid. The hygromycin-resistant colonies were grown for ten generations in the absence of hygromycin and kanamycin to allow excision of the hygromycin cassette and the loss of pJV53. Finally, unmarked Δ*greA* strain was confirmed by PCR and sequencing.

For the complementation strain of *M. smegmatis* (*comp-*Δ*greA*) and *M. tuberculosis* H37Ra (*comp-*Δ*greA*), *greA* from *M. smegmatis* strain and *greA* from *M. tuberculosis* H37Ra strain was cloned into a pMV306-P_hsp__60_ plasmid, and then the recombinant plasmid pMV306-P_hsp__60_-*greA* was transformed into *M. smegmatis* Δ*greA* and *M. tuberculosis*Δ*greA* strains, resulting in *M. smegmatis comp-*Δ*greA* and *M. tuberculosis comp-*Δ*greA*, respectively. The *comp-*Δ*greA* strains were selected on Middlebrook 7H10 agar medium (complemented with 10% OADC) containing 30 μg/ml kanamycin.

The open reading frame (ORF) of *greA* fused with 2 × FLAG was amplified on a template of *M. smegmatis* mc^2^ 155 (WT) chromosomal DNA using primer pairs. The two single-point mutants of *greA* were generated using overlapping PCR. All of the *greA* variants were cloned into a pMV306-P_hsp__60_ plasmid. The *glgX (MSMEG_3186)* gene included its native promoter were cloned into pSMT3-LxEGFP vector in order to perform flow cytometry experiment. As a result, all the plasmid constructs were confirmed via direct DNA sequencing and PCR assay.

### Construction of CRISPR Interference (CRISPRi) Targeting Constructs in *M. smegmatis*

CRISPRi targeting constructs in *M. smegmatis* was performed as previously described (Singh et al., 2016). Briefly, we have designed oligos to target two sites in the *glgX* gene. The oligos were annealed to generate a double-stranded insert which was phosphorylated by T4 polynucleotide kinase and then ligated into the *Bbs*I digested pRH2521 to obtain pRH2521-sgRNA. pRH2521 containing sgRNA-targeting the genes of interest was transformed into *M. smegmatis* competent cells into which the *dcas9*-containing vector pRH2502 was previously introduced. Transformants were selected for hygromycin and kanamycin resistance. Addition of anhydrotetracycline (ATc) was repeated every 48 h to maintain induction of *dcas9* and sgRNA for experiments.

### Antimicrobial Sensitivity Assays

Antimicrobial sensitivity assays for *M. tuberculosis* have been performed as previously described (Goodsmith et al., 2015). Mycobacteria strains were grown to early log phase and were diluted to an optical density of 0.02 in 7H9 media containing 0.05% Tween 80 and 10% OADC. Then the bacterial strains were exposed to two-fold dilutions of the following antimicrobials; rifampicin, vancomycin, bedaquiline, streptomycin, ofloxacin, isoniazid, Capreomycin, amikacin, and ethambutol (Sigma-Aldrich, United States). The minimum inhibitory concentrations (MICs) were recorded as the minimum concentrations at which the growth was inhibited by at least 90%, as compared to a control containing no antibiotic.

For *M. smegmatis*, the MICs at which no bacterial growth was observed were recorded after 3–5 days of incubation. For *M. tuberculosis*, MICs at which no bacterial growth was observed were recorded after approximately 2 weeks of incubation.

### Growth Inhibition Test

*Mycobacterium smegmatis* had been grown to early log phase and was diluted to an optical density of 0.02 in 7H9 broth medium containing 10% OADC. Then, *M. smegmatis* culture was mixed with each antibiotic, at the final concentration to 1/100th the MIC, including streptomycin, and rifampicin. The OD_600_ value of the bacteria was monitored hourly.

### Analysis of *in vitro* Response to Adverse Stress

Exponentially growing bacterial cultures (OD_600_ 0.4–0.6) were pelleted and washed three times with 7H9/Tween-80.

In *M. smegmatis*, for the low pH stress, the strains were washed twice in 7H9 acidified to pH 4.5 with HCl, supernatants were adjusted to an OD600 = 0.5, and CFU was determined after 24 h. For heat shock stresses, *M. smegmatis* strains were incubated at 48°C in a water bath for 6 h, CFUs were counted after 3∼4 days of incubation. For other stresses, *M. smegmatis* strains prepared as above were adjusted to OD600 = 0.5, and SDS, H_2_O_2_, or antibiotics were added, then the CFUs were determined after 24 h.

For *M. tuberculosis* H37Rv strains, the cells were treated only in 7H9 broth medium with 0.5% SDS and were incubated for 24 h. Then bacterial cells were diluted 10-fold and spotted on Middlebrook 7H10 agar media. While for *M. tuberculosis* H37Ra strain, the cells were resuspended in Middlebrook 7H9 broth under 0.5% SDS and were incubated for 8 h. The CFUs were counted after 4 weeks of incubation.

### Synthesis of cDNA and RT-PCR

Equal amounts of bacteria of *M. smegmatis* strains, which were grown to logarithmic phase, were collected. Total RNA and cDNA from *M. smegmatis* were extracted as previously described (Rustad et al., 2009). cDNA was prepared and amplified as previously described (Alland et al., 1998). Control reactions were performed using primers specific to *sigA* (Manganelli et al., 1999) (see Supplementary Table S1). PCR products were separated on 2% agarose gels. The mean intensity of each band was analyzed using ImageJ software. The relative expression was calculated by normalizing the intensity of the *glgX* band with that of the *sigA* band amplified with the same cDNA sample.

### Cell Culture

Murine macrophage-like RAW264.7 cells (ATCC; TIB-71) were cultured on Dulbecco’ s Modified Eagle Medium (DMEM) supplemented with 10% fetal bovine serum (FBS), 100 U/ml penicillin, and 100 μg/ml streptomycin (GIBCO, Invitrogen) with 5% CO_2_ at 37°C.

### Macrophage Infection Study

RAW264.7 cells were seeded at 3 × 10^5^ cells/well in a 24-well plate and incubated for 18 h. Homogenized bacterial suspension at a multiplicity of infection (MOI) of 10, was transformed into adherent RAW264.7 cells. The extracellular bacteria were removed by washing three times with phosphate buffer saline (PBS) after incubation for 2 h. Cells were further incubated for 48 h with 5% CO_2_. Then cell lysates were serially diluted 10-fold and spotted on 7H10-OADC agar. CFUs of *M. smegmatis* were counted after 3∼4 days of incubation at 37°C, while CFUs of *M. tuberculosis* were counted after 4 weeks days of incubation at 37°C.

### Structural Modeling

The architecture of GreA in full length was modeled with Swiss-Model^2^. The GreA of *E. coli* [PDB: 1grj.1] has functioned as a structural template, and the ribbon structure was presented with the PyMol software.

### RNA Preparation and RNA-Seq Analysis

Wild-type *M. smegmatis* mc^2^155 as well as the *greA* deletion strain were grown to the log-phase (OD600, 0.4) in Middlebrook 7H9-ADC. Total RNA was prepared from wild-type and mutant strains using TRIzol reagent (Invitrogen), followed by DNase I treatment. Approximately 1-μg total RNA samples were treated by the Ribo-Zero rRNA removal procedure (Illumina) to enrichment for mRNA. Approximately 1 μg of RNA was used for library preparation using a ScriptSeq (v2) RNA-seq kit and high-throughput sequencing on an Illumina NextSeq platform. All raw sequence reads by RNA-Seq were initially pre-processed by Trimmomatic (v0.36) to trim the adaptor sequences and remove low-quality sequences. The remaining clean reads were mapped to the *M. smegmatis* mc^2^ 155 genomes using Tophat2. The alignment results were input into Cufflinks (v2.2.1). Unless otherwise stated, the unit of expression level in our analyses is FPKM. Cuffdiff (v2.2.1) was used to test for differential expression. We defined genes as differentially expressed using the following criteria: FPKM > 10 and FDR < 0.001. The data for visualization was generated by R (R Development Core Team, Vienna, Austria). The genome sequences and the annotations of *M. smegmatis* mc^2^ 155 were obtained from the GenBank^2^. We analyzed differences in the enrichment of Gene Ontology (GO) categories and KEGG pathways for the DEGs using the DAVID (v6.8) functional annotation analysis tool^3^.

### Chromatin Immunoprecipitation

ChIP was performed using log-phase (OD600, 0.4) liquid medium grown cultures of *M. smegmatis* strains producing GreA-FLAG. As a negative control, WT log-phase culture was used. The cells were fixed with 1% formaldehyde for 30 min, the reaction was quenched with 150 mM glycine for 15 min, and then the cells were washed three times with cold PBS (54,000 rpm, 5 min, 4°C) and frozen at −80°C. To prepare lysates, pellets were resuspended in FA-1 buffer (HEPES-KOH at 50 mM [pH 7.5], NaCl at 140 mM, EDTA at 1 mM, Triton X-100 at 1%, and protease inhibitor cocktail [Sigma-Aldrich]), disintegrated with silica beads (0.1 mm) for 45 min, and sonicated on ice using ten cycles of a 10-s pulse followed by a 50-s pause. The obtained lysates were centrifuged (5 min, 12,000 rpm, 4°C) and frozen at −80°C in 5% glycerol. For immunoprecipitation, 200 μg of total protein was incubated on a rotary shaker (Thermo Fisher Scientific, China Co., Ltd.) for 4 h at 4°C with a 15-μl packed-gel volume of anti-FLAG M2 magnetic beads (Sigma-Aldrich) and then washed twice with FA-1 buffer. Samples were processed in a final volume of 0.5 ml in two biological replicates, with input DNA controls (200 μg of total protein alone) included for each replicate. Samples were washed using a magnetic separator with sequential applications of FA-1 buffer, FA-2 buffer (HEPES-KOH at 50 mM [pH 7.5], NaCl at 500 mM, EDTA at 1 mM, Triton X-100 at 1%, and protease inhibitor cocktail), and Tris–EDTA buffer solution (TE) (Tris–HCl at 10 mM [pH 8.0], EDTA at 1 mM). Immunoprecipitated samples were de-cross-linked overnight in TE containing 1% SDS at 65°C and then digested with proteinase K (final concentration, 0.05 mg/ml) for 1.5 h at 55°C. The immunoprecipitated DNA was extracted using Cycle-Pure Kit (OMEGA Bio-tek, Inc.).

### Library Construction and Illumina Sequencing

The library of DNA fragments was prepared using a QIAseq Ultralow Input library kit (Qiagen). Briefly, the protocol includes DNA end-repair, sequencing adapter ligation, cleanup, and PCR amplification. At the end of the procedure, quantification and quality evaluations were done using a NanoPhotometer ^®^ spectrophotometer (IMPLEN), a Qubit^®^ 2.0 Fluorometer (Life Technologies), and a 2100 Bioanalyzer (Agilent). Second-generation sequencing was performed using a HiSeq 1500 sequencing platform (Illumina).

### Analysis of CHIP-Seq Data

The obtained FASTQ files were filtered according to read quality, and adapter sequences were trimmed using the Trimmomatic software (Usadel Lab; Aachen University, Aachen, Germany). The filtered FASTQ files were mapped to the genome of *M. smegmatis* strain mc^2^ 155 (from GenBank) using the BWA v0.7.12 (Burrows-Wheeler aligner), and the Bwa mem algorithm was applied. The bam files were sorted and indexed. Then the bam files for the ChIP and input samples were subjected to MACS analysis (MACS2 software) for ChIP-Seq peak detection. Peak calling was performed without building a model using a shift size of 150 bp. The ChIP-Seq peaks were uploaded into the R environment as bed files, and the peaks were annotated ChIP-Seq data.

### Statistical Analysis

Statistical analysis was performed using Prism (version 6.0c; GraphPad Software). Data were analyzed using the paired Student’s *t*-test, and in the comparisons of data from three or more conditions, analysis of variance (ANOVA) was used. A *p*-value of 0.05 or less was considered significant.

## Results

### Mutants for *greA* Caused a Delay in Mycobacterial Growth

**FIGURE 1:**
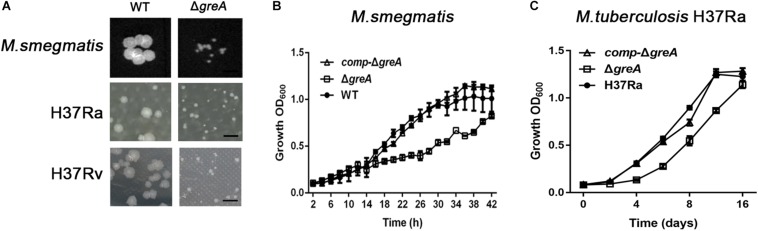
Mutants for *greA* on growth, colonial morphology in mycobacteria. **(A)** The colony size of WT and Δ*greA* strains from *M. smegmatis*, H37Ra and H37Rv. The bar corresponds to 0.5 cm. The growth of WT, Δ*greA*, and *comp-*Δ*greA* strains from **(B)** *M. smegmatis* and **(C)** *M. tuberculosis* H37Ra in 7H9 broth. Bacteria were diluted to an initial OD_600_ of 0.02 and the OD_600_ was monitored at the designated time points. Data are shown as mean ± SEM of three independent experiments.

To explore the function of *greA* in mycobacteria, we deleted the *greA* (*MSMEG_5263*) gene by allelic exchange facilitated via recombination in *M. smegmatis* mc^2^155 which is widely used as a model to study non-pathogenic aspects of mycobacterial physiology. We have detected colonies of small sizes in the *greA* mutant strain compared with the parental strain (Figure 1A), it was observed that Δ*greA* strain grows slower than the wild-type *M. smegmatis* and the phenotypes that were complemented by expression of *greA* (*comp*-Δ*greA*) from *M. smegmatis* on the chromosome (Figure 1B). To confirm the phenotype of GreA in *M. tuberculosis* strain, we knocked out *greA* (*rv1080c*) in *M. tuberculosis* H37Rv and H37Ra by specialized transducing mycobacteriophages (Supplementary Figure S1). Following the observation that a growth defect was caused by *greA* deficiency in *M. smegmatis*, we found that colonies of small sizes were also observed in the Δ*greA* strain of H37Ra and H37Rv (Figure 1A) and a growth defect was detected in the Δ*greA* strain of H37Ra (Figure 1C). Our observation was consistent with a previous study (China et al., 2011).

### Deletion of *greA* Increased the Sensitivity to Adverse Stress

**FIGURE 2:**
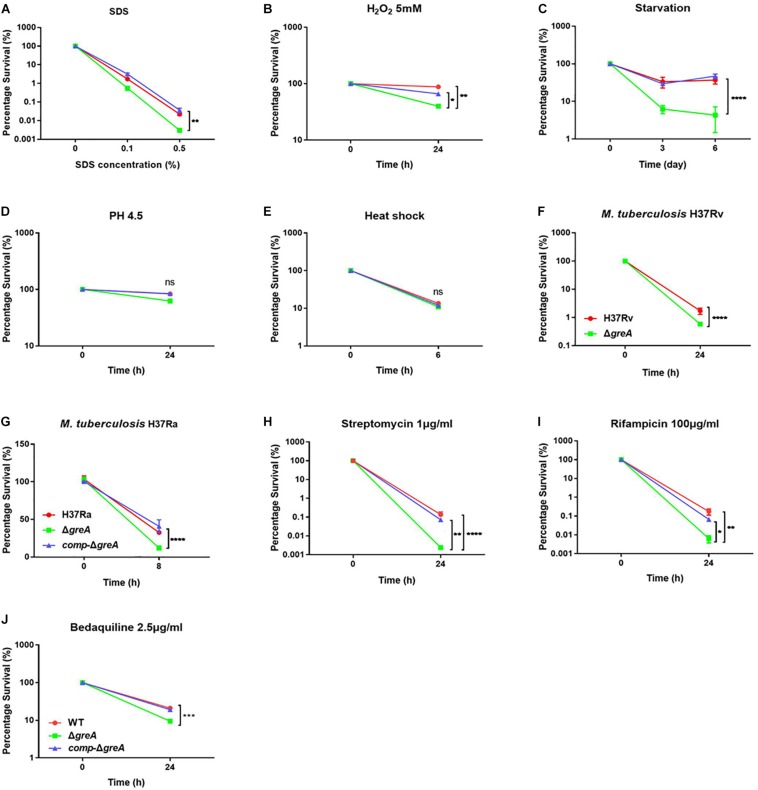
GreA is required for resistance to adverse stress. **(A–E, H–J)** Survival of *M. smegmatis* strains during adverse stress. The strain symbols listed in the **(J)**. WT, Δ*greA* and *comp-*Δ*greA* strains were treated for at the indicated time with varying concentrations of SDS **(A)**, 5mM H_2_O_2_ **(B)**, PBS + 0.05% Tween 80 **(C)**, low pH (pH4.5) **(D)**, heat shock (48°C) **(E)**, 1 μg/ml of streptomycin **(H)**, 100 μg/ml of rifampicin **(I)**, or 2.5 μg/ml of bedaquiline **(J)**. After treatment, dilutions were plated on 7H10 and survival is expressed as a ratio of CFUs compared to untreated cultures. **(F)** *M. tuberculosis* H37Rv strains were spotted on the 7H10 plates after they were treated under 0.5% SDS for 24 h. **(G)** WT, Δ*greA*, and *comp-*Δ*greA* strains from *M. tuberculosis* H37Ra were subjected to 0.5% SDS for 8 h. CFU enumeration was performed after incubation for the indicated time. These experiments were performed three times with similar results. Data are shown as mean ± SEM of three independent experiments. *p < 0.05, **p < 0.01, ***p < 0.001, ****p < 0.0001, ns, *p* ≥ 0.05.

It has been reported that GreA is essential for the survival of *E. coli* under stress conditions (Sharma and Chatterji, 2010). Herein, we investigated the role of GreA in the environmental adaptation of mycobacteria. The WT, Δ*greA*, and *comp*-Δ*greA* strains were treated with adverse stresses. When compared to parental bacteria and complement strain, the Δ*greA* strain was more sensitive to SDS, hydrogen peroxide, and starvation (Figures 2A–C). However, there were no differences in the survival rate of bacteria which were treated with low pH and heat shock (Figures 2D,E). In addition, we also found that the survivability of *M. tuberculosis* Δ*greA* mutant strain was significantly reduced compared to H37Rv strain upon the treatment with 0.5% SDS (Figure 2F) and a similar phenotype was also observed in *M. tuberculosis* H37Ra strain (Figure 2G). Moreover, the *greA* gene also complemented the phenotype of Δ*greA* in *M. tuberculosis* H37Ra (Figure 2G). These results indicated that mycobacteria GreA is required for adaptation to these stressful conditions.

Of note, the *greA* gene was found upregulated under stress with antibiotics in *M. tuberculosis* (Sharma et al., 2010; Lata et al., 2015). We speculated that Δ*greA* strain might be more susceptible to antibiotics. To test this hypothesis, we performed antibiotic susceptibility tests where, *M. smegmatis* WT, Δ*greA*, and *comp-*Δ*greA* strains were challenged with streptomycin, rifampicin, and bedaquiline. We found that Δ*greA* strain was more susceptible to the tested antibiotics compared with both parental and complemented bacteria (Figures 2H–J). Moreover, we also evaluated the susceptibility to antibiotics through the MIC test in *M. tuberculosis*, and we found that *M. tuberculosis*Δ*greA* strain showed increased susceptibility to rifampicin, bedaquiline, and vancomycin. Surprisingly, the MIC of the Δ*greA* strain decreased by 4-fold with rifampicin and by 8-fold with vancomycin compared with MTB H37Rv (Table 1), suggesting that *greA* plays an important role in the antibiotic susceptibility in mycobacteria.

**TABLE 1:** Changes of MICs of wild-type mycobacteria and Δ*greA* strains against different antimicrobial agents.

**Mycobacteria strains**	**Minimum inhibitory concentrations (mg/L)**								
	**INH**	**RIF**	**EMB**	**SM**	**CPM**	**OFX**	**VAN**	**AMK**	**BDQ**
H37Rv	0.0125	0.018	1	2.5	5.12	0.32	6.25	5.12	0.05
Δ*greA*-H37Rv	0.0125	0.0045	1	2.5	5.12	0.32	0.781	5.12	0.025
mc^2^155	8	16	1	0.5	5	1	10	0.5	0.05
Δ*greA*-mc^2^155	8	16	1	0.25	1.25	1	5	0.25	0.025
*comp-*Δ*greA*-mc^2^155	8	16	1	0.5	5	1	10	0.5	0.05
H37Ra	0.0375	0.0015	1	0.1	5.12	0.32	3.125	5.12	0.02
Δ*greA*-H37Ra	0.0375	0.0002	0.5	0.1	5.12	0.32	0.781	5.12	0.01
*comp-*Δ*greA*-H37Ra	0.0375	0.0015	1	0.1	5.12	0.32	3.125	5.12	0.02

*VAN, vancomycin; INH, isoniazid; RIF, rifampicin; EMB, ethambutol; OFX, ofloxacin; AMK, amikacin; SM, streptomycin; CPM, capreomycin; BDQ, bedaquiline.*

In accordance with the observation that the *greA* mutant strain has an increase in the sensitivity to antibiotics, we found that the growth of Δ*greA* strain was more retarded despite at low antibiotic concentrations used when compared to WT and complemented strains (Supplementary Figure S2).

### *In vivo* Transcription Analysis of GreA-Responsive Genes

**FIGURE 3:**
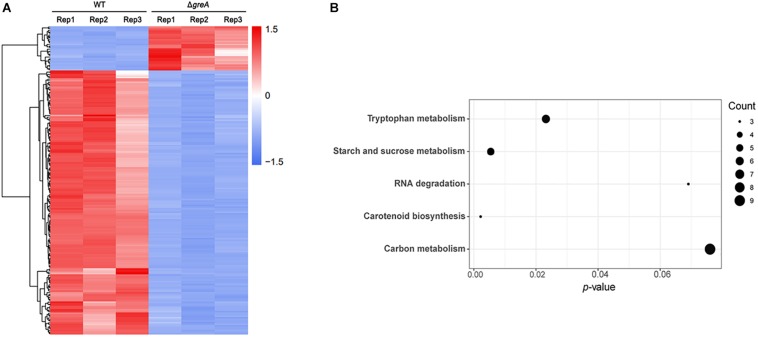
Differentially regulated genes were identified between Δ*greA* strain and WT strain by RNA-seq. **(A)** Heat maps showing the expression profiles of genes upregulated or downregulated by deletion of the *greA* gene in *M smegmatis*. **(B)** Summary of the KEGG reference pathway results. Three biological replicates were tested.

Given our observations that inactivation of *greA* in *M. smegmatis* results in reduced survivability under stress, we next sought to identify the proteins being regulated by GreA. To understand how GreA may affect gene expression at the transcriptional level, we performed whole-transcriptome RNA-seq analyses. We found that disrupting *greA* resulted in the differential regulation of 195 genes in *M. smegmatis* during the exponential growth. Among these, 28 were positively regulated and 167 were negatively regulated (Figure 3A and Supplementary Table S2). Moreover, our findings for enriched KEGG pathways are summarized in Figure 3B and Supplementary Table S3. Three pathways were significantly overrepresented. Three genes are involved in tryptophan metabolism, and four genes are involved in starch and sucrose metabolism. Interestingly, the *glgX* gene, which encodes the debranching enzyme of the catabolic enzymes, was involved with starch and sucrose metabolism (Schneider et al., 2000). Chromosomal inactivation of *glgX* in *Corynebacterium glutamicum* led to slower growth (Seibold and Eikmanns, 2007), it is suggested that GreA mediated regulation of the growth might be partly associated with *glgX*. This outcome demonstrated that GreA has a profound effect on cellular gene regulation, especially on metabolic associated genes. Collectively, these transcriptional changes are consistent with the scenario that *greA* gene expression is required for optimal growth of mycobacteria, whereas loss of *greA* results in slower growth and reduced tolerance to adverse environments.

### Identification of GreA-Binding Sites Using ChIP-Seq

**FIGURE 4:**
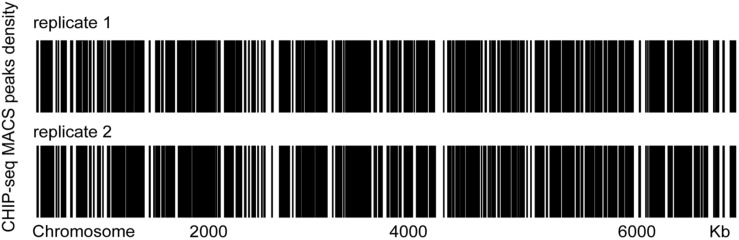
Heat maps of the distributions of GreA-FLAG binding sites in two biological replicates. Two biological replicates were tested.

To examine whether GreA binds to specific chromosomal regions, we performed chromatin immunoprecipitation followed by deep sequencing (CHIP-seq) and then sought to verify the binding mode of GreA. CHIP-seq experiments were performed by using *M. smegmatis* strains producing GreA proteins fused with two repeats of the FLAG epitope (FLAG_2_). GreA-FLAG_2_-DNA nucleoprotein complexes fixed with formaldehyde in the log phase of growth were immunoprecipitated using magnetic beads. To exclude unspecific interactions with magnetic beads, we used a WT strain lacking the FLAG epitope as a negative control for our CHIP-seq experiments. Enriched regions were determined by comparison to the background noise level, which was estimated versus the input DNA of each ChIP-Seq replicate. Using the data obtained from this analysis, we established chromosomal binding maps for the analyzed proteins. The GreA-FLAG_2_ binding sites were distributed evenly along the *M. smegmatis* (Figure 4). The identified GreA-FLAG_2_ binding sites included 1005 CHIP-seq peaks that were confirmed in two biological replicates and absent in WT strain (Supplementary Table S4). Among the 1005 CHIP-seq peaks identified for GreA-FLAG_2_, we observed that several binding sites were located downstream of the gene (∼0.7% of all ChIP-Seq peaks). However, GreA binding sites seemed to be more frequent within promoters (∼84.8% of all ChIP-Seq peaks), and ∼14.5% of all ChIP-Seq peaks were identified in gene bodies (Supplementary Figure S3). Notably, from the CHIP-seq dataset, we found that one of the peaks was located at the coding sequence of *glgX*, suggesting that GreA might regulate the transcription of *glgX*.

### The Full Activity of the GreA Is Crucial in *M. smegmatis* Growth

**FIGURE 5:**
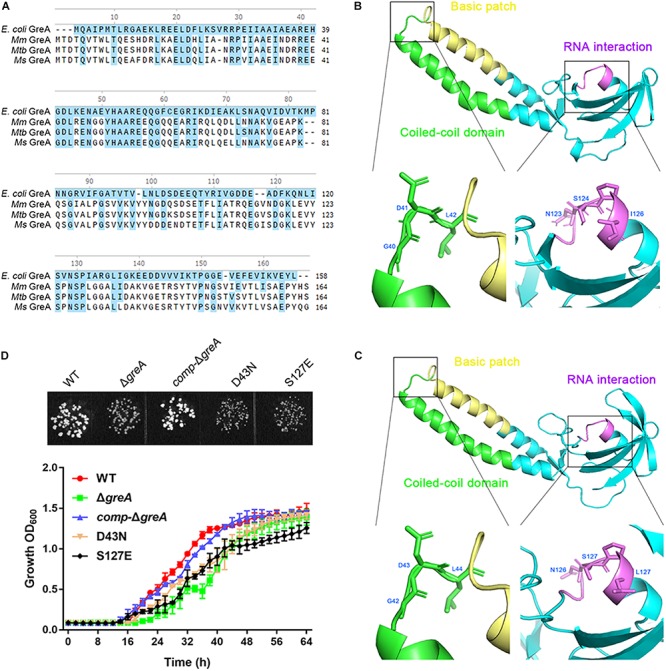
Optimal growth of *M. smegmatis* is required for the full activity of the GreA. **(A)** Multiple sequence alignment of *E. coli* GreA (158 aa) with *M. smegmatis* GreA (164 aa), *M. tuberculosis* GreA (164 aa), and *M. marinum* (165 aa). The conservative residues of *E. coli* GreA **(B)** and *M. smegmatis* GreA **(C)**. PyMol is applied to generate the photographs of coiled-coil domain, basic patch, and RNAP interaction. **(D)** The growth of *M. smegmatis* strains harboring *greA* and/or its point mutants. Three biological replicates were tested. Data are shown as mean ± SEM of three independent experiments.

It is known that *E. coli* GreA consists of two domains: an N-terminal extended coiled-coil domain (NTD, residues 1–75) and a C-terminal globular domain (CTD, residues 76–158) (Stebbins et al., 1995; Koulich et al., 1997). The NTD carries the structural determinants conferring the cleavage activity and, presumably, the anti-arrest and readthrough activities. The CTD participates in binding to RNAP (Koulich et al., 1998). Alignment of the mycobacterial GreA with *E. coli* GreA revealed the similar conserved features (Figure 5A). In addition, GreA from *M. smegmatis* is almost identical to that of *M. tuberculosis* GreA, indicating its similar function in mycobacteria. To gain further structural insight into the conserved domain of mycobacterial GreA, structural modeling by the Swiss-Model program was performed using *E. coli* GreA (PDB accession no. 1grj.1) as a structural template (Figure 5B). The ribbon structure of *M. smegmatis* GreA, together with *E. coli* GreA were generated with the PyMol software. Of note, structural comparison of the two GreA proteins illustrated that the coiled-coil domain and RNAP interaction of CTD constitute almost identical motifs (Figure 5C). To further explore their physiological role in the growth phenotype of GreA, we applied overlapping PCR to generate the following two single point mutations of GreA (D43N, S127E). As expected, the D43N and S127E mutations completely disrupted the optimal growth of *M. smegmatis* GreA (Figure 5D), the observation is in line with a previous *in vitro* study (China et al., 2011). Collectively, these observations strongly support the conclusion that the full activity of the GreA is required for *M. smegmatis* growth.

### The *glgX* Gene Regulated by GreA Is Involved in the Optimal Growth of *M. smegmatis*

**FIGURE 6:**
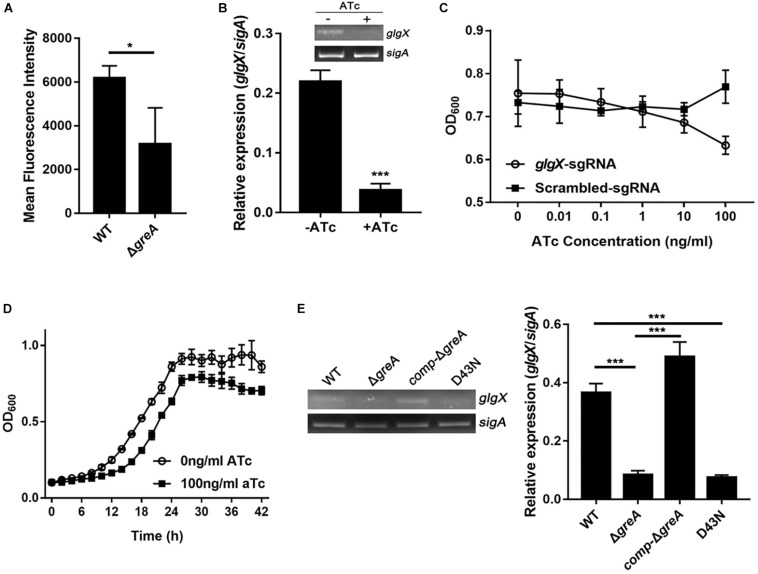
The *glgX* gene regulated by GreA is involved in the optimal growth of *M. smegmatis*. **(A)** Bacteria was collected at logarithmic phase. The mean fluorescence intensity of GFP in the *M. smegmatis* strains harboring a plasmid encoding *gfp* fusion with the *glgX* gene including its native promoter. **(B)** Expression of *glgX* was determined by semi-quantitative RT-PCR with or without induction of ATc. **(C,D)** *In vitro* growth of culture with or without induction of ATc. **(E)** Results of semi-quantitative RT-PCR of glgX in *M. smegmatis* strains harboring *greA* and/or its point mutants. **(B,E)** The gel images shown are representative of three repeat experiments. The PCRs of *sigA* served as controls for normalization of the cDNAs. The relative expression of *glgX* versus that of *sigA* was quantitated using ImageJ. The bar graphs show the means of two independent experiments. The error bars indicate the standard errors of the means (SEM). Three biological replicates were tested. *p ¡ 0.05, ***p < 0.001.

To extend our *in silico* finding, we performed genetic experiments to confirm the relationship between GreA and a candidate gene. We selected the candidate gene based on their down-regulation in the RNA-seq data, the enrichment in CHIP-seq data, and the potential role of their gene product in the growth phenotype. Taking these into consideration, we focused on the function of the *glgX* gene. To determine whether the reduced growth of *M. smegmatis greA* mutant strain was attributed to the decreased expression of *glgX*, we applied the mycobacterial CRISPRi for rapid validation. Initially, we introduced a plasmid encoding the gene of green fluorescent protein (GFP) fused with the *glgX* gene, including its native promoter, into both wild type and mutant *M. smegmatis* strains. Then we determined the mean fluorescence intensity of GFP by the flow cytometry. The mean fluorescence intensity of GFP was significantly decreased in Δ*greA* strain compared to WT strain (Figure 6A), confirming that disruption of *greA* was responsible for the reduced expression of *glgX*. Moreover, the silencing efficiency was measured by semi-quantitative RT-PCR that showed a ∼80% decrease in the expression of the *glgX* gene (Figure 6B). As expected, the decrease in the expression of *glgX*, resulted in reduced growth compared to the strain without induction of ATc (Figures 6C,D), indicating that loss of *greA* has led to a partial defect of growth by down-regulating *glgX*.

In *E. coli*, the residues D41 of the GreA protein are required to prevent the backtracking of paused complexes, thereby suppressing the transcriptional pauses (Vinella et al., 2012). Since the GreA protein of *E. coli* and *M. smegmatis* shares the residues mentioned above, we determined if the anti-backtracking activity of GreA was associated with the regulation *glgX*. To achieve this, the transcriptional expression of *glgX* was detected in *M. smegmatis* strains. Interestingly, transcriptional expression of *glgX* was fully restored in *comp-*Δ*greA* strain. However, this has not been observed in the D43N strain and *greA* mutant strain (Figure 6E), indicating that the anti-backtracking activity of GreA is required for the transcriptional expression of the *glgX* gene.

### Deletion of *greA* Attenuated the Intracellular Survivability

**FIGURE 7:**
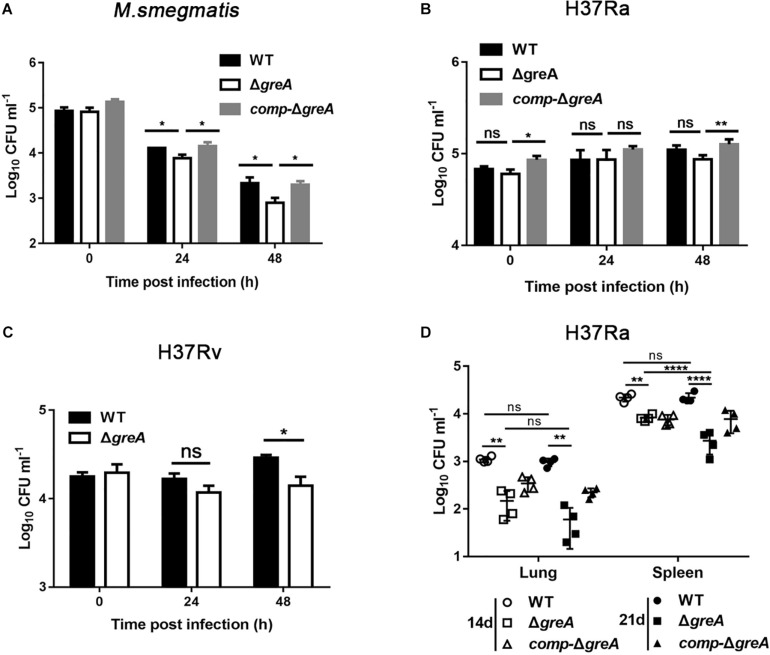
Intracellular fitness of Δ*greA* mutant strain was evaluated *in vivo* and *in vitro*. RAW264.7 cells were seeded at 3 × 10^5^ cells per well in 24-well culture plates. After adhesion, cells were infected with bacteria at MOI of 10. The extracellular bacteria were removed 2 h post infection by washing with PBS. The cells of *M. smegmatis* **(A)**, *M. tuberculosis* H37Ra **(B)**, and *M. tuberculosis* H37Rv **(C)** were further incubated for 24 and 48 h at 37°C. Then serially diluted (10-fold) cells lysates were spotted on a 7H10-OADC agar and cultured in 37°C. CFUs were counted after incubation for the indicated time for *M. smegmatis* and *M. tuberculosis*, respectively. **(D)** Mice were infected i.p. (1 × 10^7^) with WT, Δ*greA*, or *comp-*Δ*greA* strains of *M. tuberculosis* H37Ra. The number of surviving bacilli by CFU counting at the indicated time. Data are shown as mean ± SEM of two independent experiments. *p < 0.05, **p < 0.01, ****p < 0.0001, ns, *p* ≥ 0.05.

The persistence inside a host macrophage is a vital characteristic that distinguishes the pathogenic mycobacteria from non-pathogenic strains (Singh et al., 2008; Yaseen et al., 2015). RAW264.7 cells were infected with WT, Δ*greA* or complemented strains of *M. smegmatis*. The numbers of the surviving intracellular bacteria were assessed after 24 and 48 h post-infection. A significant reduction in the number of viable intracellular mycobacteria was observed after 24 and 48 h in the Δ*greA* strain (Figure 7A). Moreover, the survivability of H37Rv and Δ*greA* strain was assessed by the detection of the bacterial CFUs inside the macrophage. We found that the survivability of *greA* mutant strain was significantly decreased in macrophages after 48 h when compared to the H37Rv strain (Figure 7C). In addition, the RAW264.7 cells were infected with WT, Δ*greA*, or complemented strains of *M. tuberculosis* H37Ra. In this case, no significant difference between H37Ra and Δ*greA* strain for intracellular survivability was observed at 48 h post-infection (Figure 7B). The findings lead us to suppose that this survival phenotype may be the result of the growth defect of *M. tuberculosis* lacking *greA*. It has been demonstrated that strain H37Ra is avirulent, and does not multiply in mice (Larson and Wicht, 1964). Thus, we believe that *in vivo* experiments may help to clarify this ambiguous phenomenon. To do this, mice were infected with WT, Δ*greA*, or complemented strains of *M. tuberculosis* H37Ra. After 14-days of infection, we observed a decrease of bacterial burden in mice infected with the H37Ra *greA* mutant strain (Figure 7D).

Overall, these results emphasize that GreA facilitates the intracellular survival of mycobacteria, and thus revealed its critical role in both an *in vitro* and an *in vivo* infection model.

## Discussion

### GreA Is Essential for Sustaining Mycobacterial Growth and Environmental Adaptation

Few studies are available about the functional characteristics of GreA in *Mycobacterium* spp. A previous study showed that knocking down of *greA* resulted in growth retardation in *M. tuberculosis* H37Ra (China et al., 2011). For example, the absence of *greA* in *S. pneumoniae* severely perturbed its growth (Yuzenkova et al., 2014). Regarding the universal role of Gre factors in prokaryotes, it is not surprising that deletion of *greA* in *M. tuberculosis* resulted in growth retardation.

It has been convincingly shown in several studies that GreA is required for the survival of bacteria under stress. For instance, Wei and colleagues found that the deletion of *greA* in *Sinorhizobium meliloti* results in sensitivity to salt stress (Wei et al., 2004). Another study revealed that overexpression of GreA provides the host cells with enhanced resistance to heat shock and oxidative stress in *E. coli* (Li et al., 2012). A very recent study has shown that similar with *E. coli* GreA, *M. smegmatis* GreA exhibits chaperone-like activity *in vitro*, which prevents heat-induced aggregation of substrate protein (Joseph et al., 2019). Intriguingly, it has been established that double-deletion of *greA* and *greB*, encoding a transcript cleavage factors function-similar to that of GreA, causes heat sensitivity, indicating that another Gre factor may partially complement the phenotype of *greA* mutant. Our data shows that GreA is dispensable for the survival of *M. tuberculosis* under heat shock stress. It seems to be that the phenotype, in part, may be complemented by another copy of *greA*, since there are two genes (*MSMEG_5263* and *MSMEG_6292*) encoding transcription elongation factor GreA in *M. smegmatis* (Mohan et al., 2015). Further experiments are required to address the role of MSMEG_6292 in *M. smegmatis*.

Moreover, the deletion of *greA* in *M. smegmatis* increased the susceptibility to bedaquiline, capreomycin, streptomycin, amikacin, and vancomycin (Table 1). Notably, our data has established that the growth defect of Δ*greA* strain was more obvious despite the low antibiotic concentrations used. This finding is crucial, as it provides the evidence to support that a GreA inhibitor might be able to enhance the efficacy of the current anti-TB drugs.

### Transcription Regulation by the *M. smegmatis* Transcription Elongation Factor GreA

Here, we showed that 167 genes were down-regulated in the *M. smegmatis* lacking the *greA*, while only 28 genes were positively regulated. Most of the down-regulated genes were associated with metabolism, supporting the view that GreA optimizes mycobacterial growth and environmental adaptation by the regulation of metabolism. For example, *glgX*, a gene involved in starch and sucrose metabolism, was significantly down-regulated in *greA* mutant strain and the inactivation of *glgX* in *C. glutamicum* led to slower growth (Seibold and Eikmanns, 2007). It is suggested that GreA mediated regulation of the growth might be partly associated with *glgX*. In addition, *katG*, a gene encoded catalase-peroxidase-peroxynitritase, plays a role in the intracellular survival of the mycobacteria within macrophages and in protection against reactive oxygen and nitrogen intermediates produced by phagocytic cells (Milano et al., 2001; Pym et al., 2002; Sassetti et al., 2003). Given the down-regulation of *katG* in the *greA* mutant strain, this could explain the role of GreA in mediating the regulation of oxidation resistance.

GreA has an impact on the regulation of gene expression in *E. coli* (Roberts et al., 2008) and *S. pneumoniae* (Yuzenkova et al., 2014) by affecting the transcription elongation. In addition to the effect of GreA on transcription elongation, competition between GreA and DksA has been reported. DksA, an RNAP-binding transcription factor, binds to the secondary channel of RNAP and plays a vital role in controlling polyP activation, which is required for stress survival and virulence in diverse pathogenic microbes (Gray, 2019).

Moreover, GreA and DksA, have an opposite regulation of a subset of genes in *E. coli* (Vinella et al., 2012). These data suggest that GreA might have a global role in the regulation of genes expressed in a prokaryotic organism. From our CHIP-seq dataset, it was revealed that GreA is symmetrically distributed on the *M. smegmatis* chromosome. However, we were unable to identify discrete binding sites for GreA. Of note, there is no evidence that the Gre factors can bind with nucleic acids. Indeed, the Gre factors are proteins interacting with the RNAPs, this might suggest that GreA indirectly regulates the gene expression by interaction with the RNAP complex.

GreA consists of two domains; a C-terminal globular domain and an extended N-terminal coiled-coil domain. The NTD is responsible for the GreA induction of nucleolytic activity, while the CTD determines the binding of GreA to RNAP. Both domains are required for full functional activity of GreA (Koulich et al., 1998). We also confirmed that the full activity of the GreA is crucial in *M. smegmatis* growth. Of note, it has been reported that, another Gre homolog, Rv3788 binds near the secondary channel of RNAP to inhibit transcription. However, the factor did not show any transcript cleavage stimulatory activity (China et al., 2012). Furthermore, our data demonstrated that *glgX*, a gene involved in starch and sucrose metabolism, regulated by the anti-backtracking activity of GreA is required for the optimal growth of *M. smegmatis*. Recently, studies in *Salmonella enterica* serovar Typhimurium demonstrated that GreA-mediated rescue of backtracked paused complexes during transcription is crucial in promoting *hilD* expression through its 3′-UTR (Gaviria-Cantin et al., 2017). In addition, GreA enhanced expression of several *E. coli* RNAP promoters *in vitro* (Erie et al., 1993; Feng et al., 1994; Hsu et al., 1995; Maddalena et al., 2016). Given that GreA has a multitude of effects on transcription, initiation, and elongation (Stepanova et al., 2007; Vinella et al., 2012), this could explain our failure to identify the binding motif of GreA. Based on the present study, we suppose that the anti-backtracking activity GreA is involved in the regulation of gene expression in mycobacteria. It would be interesting in future studies to examine the regulation of GreA on gene expression *in vitro*.

It is known that GreA influences not only cellular invasion but also replication in *Francisella tularensis* subsp. *Novicida* (Cui et al., 2018) and *Salmonella typhimurium* (Gaviria-Cantin et al., 2017). Our data also revealed that *greA* deletion caused a slight reduction of intracellular fitness in *M. tuberculosis* H37Rv, suggesting that GreA is required for the maintenance of intracellular survival of *M. tuberculosis*. The use of a non-pathogenic species, *M. smegmatis*, may have been a limitation of the present study. Although there is some evidence that *M. smegmatis* has been used as a model to study the function of interesting gene of *M. tuberculosis* (Wang et al., 2015, 2017; Yaseen et al., 2015, 2018), *M. smegmatis* isn’t closely related to *M. tuberculosis* in genetically. Very recently, based on the comprehensive phylogenomic analyses and comparative genomic studies, *M. smegmatis* categorized as a novel classification of Mycobacterium is distinguished as a different genus comparing to *M. tuberculosis.* (Gupta et al., 2019). It suggests that certain functions of GreA in pathogenic *M. tuberculosis* may be different from in *M. smegmatis*, especially with the virulence, since most experiments have been performed in *M. smegmatis*, instead of *M. tuberculosis* H37Rv. In a further study, we will provide evidence to elucidate the regulation role of GreA in pathogenic *M. tuberculosis*.

In conclusion, our study revealed that GreA plays an important role in mycobacterial biology where, the deletion of *greA* resulted in bacterial growth retardation, reduction of intracellular fitness, and more importantly from a clinical treatment point of view, improvement of antibiotic susceptibility. Overall, our results strongly suggest that GreA warrants being studied as a potential target for the development of MDR-TB inhibitor therapy.

## Data Availability Statement

The datasets for this study can be found in GEO, under accession number GSE143764: https://www.ncbi.nlm.nih.gov/geo/query/acc.cgi?acc=GSE143764.

## Ethics Statement

All animal experiments were performed in accordance with the National Institutes of Health Guide for the Care and Use of Laboratory Animals, and the experimental procedures were approved by the Ethics Committee of Zhongshan School of Medicine on Laboratory Animal Care (reference number: 2016-159), Sun Yat-sen University.

## Author Contributions

GT conceived the study and supervised global data analysis. HC and AR Provided advice in design and co-edited manuscript. SF and YL Designed and performed the experiments, analyzed the data, and co-wrote the manuscript. WL and ME-S performed the construction of deletion of Gre in mycobacteria. ZhZ and CS performed the intracellular fitness experiment. ZiZ and ZG designed and performed the RNAseq experiment. LuL and LiL performed the ChIPseq experiment. YY and XW contributed to data interpretation and wrote the manuscript. All authors read and approved the final manuscript.

## Conflict of Interest

The authors declare that the research was conducted in the absence of any commercial or financial relationships that could be construed as a potential conflict of interest.

## Footnotes

1 https://www.ncbi.nlm.nih.gov/genbank/

2 https://swissmodel.expasy.org/repository/uniprot/A0R2X1

3 https://david.ncifcrf.gov

## Supplementary Material

FIGURE S1 Generation of *greA* gene replacement mutant. Schematic representation of the strategy used for the generation of *greA* gene replacement mutant and confirmation of *greA* disruption in *M. smegmatis* **(A)**, *M. tuberculosis* H37Rv **(B)**, and *M. tuberculosis* H37Ra **(C)**, respectively.

FIGURE S2 Deletion of *greA* altered susceptibility of antibiotics in *M. smegmatis*. WT, Δ*greA* and *comp-*Δ*greA* strains were exposed to 7H9 medium in the absent (control) or present of different antibiotics at 1/100th the MIC. The initial OD_600_ was 0.02. The growth curves were monitored for 42 h. **(A)** Streptomycin (MIC = 0.005 mg/L) and **(B)** Rifampicin (MIC = 0.16 mg/L).

FIGURE S3 Analysis of binding sites identified from the ChIP-seq experiments. **(A)** Histogram showing the frequency distribution of distances between GreA ChIP-seq peak centers and TSSs. **(B)** Chromosomal binding map of HupB-FLAG.

TABLE S1 All primers and strains used in this study.

TABLE S2 Transcriptome of the *M. smegmatis* wild type and the Δ*greA* mutant.

TABLE S3 ChIP-seq of GreA: ChIP-seq binding sites.

TABLE S4 Results of gene ontology analyses (DAVID; FDR < 0.05).
