# Supplementary figures and images for: Involvement of Transcription Elongation Factor GreA in Mycobacterium Viability, Antibiotic Susceptibility, and Intracellular Fitness
Source: Front Microbiol. 2020 Mar 23;11:413. doi: 10.3389/fmicb.2020.00413 (PMC7104715; doi:10.3389/fmicb.2020.00413)

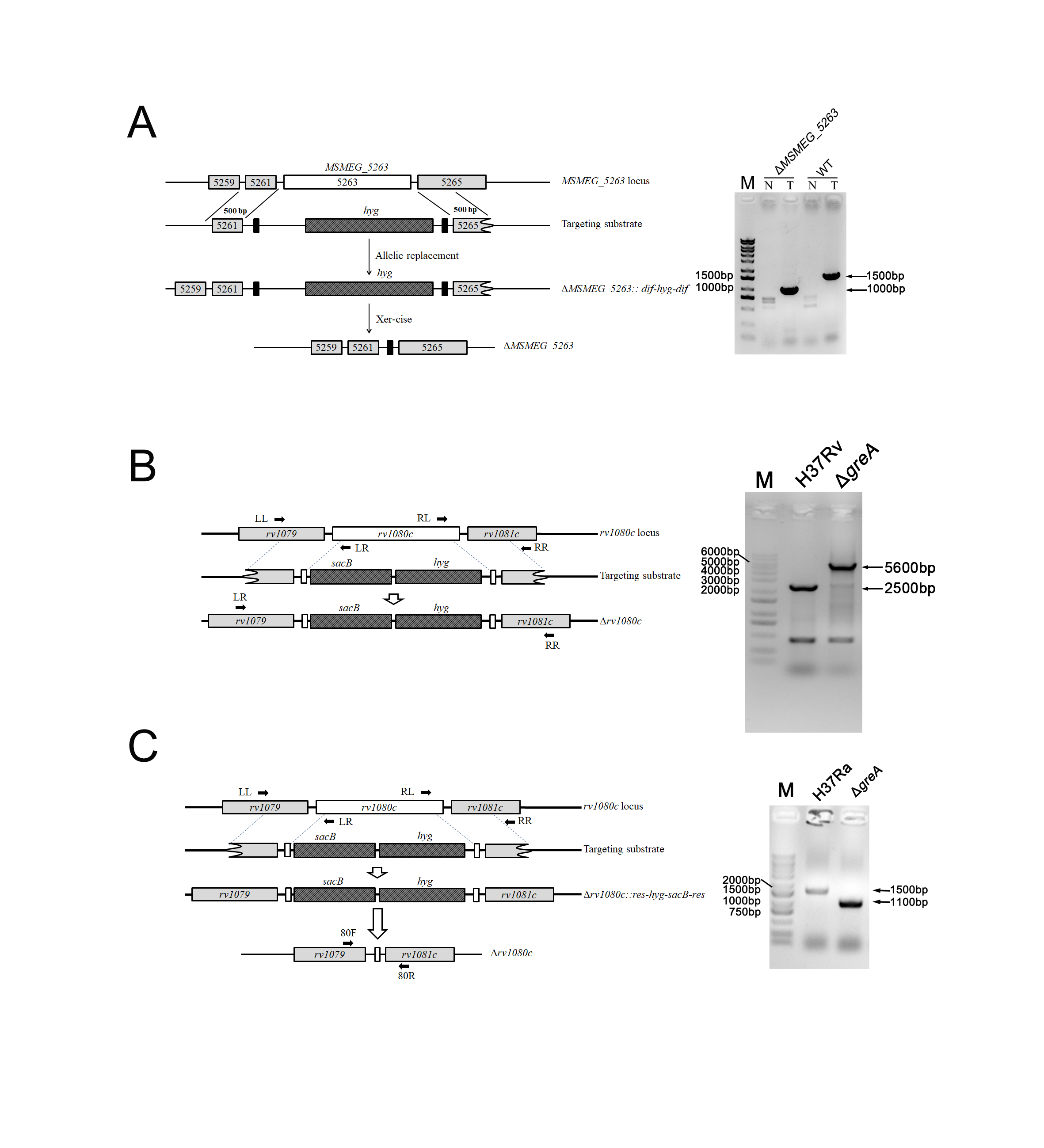

Supplement: FIGURE S1 — Generation of greA gene replacement mutant. Schematic representation of the strategy used for the generation of greA gene replacement mutant and confirmation of greA disruption in M. smegmatis (A), M. tuberculosis H37Rv (B), and M. tuberculosis H37Ra (C), respectively. [file Image_1.TIF]

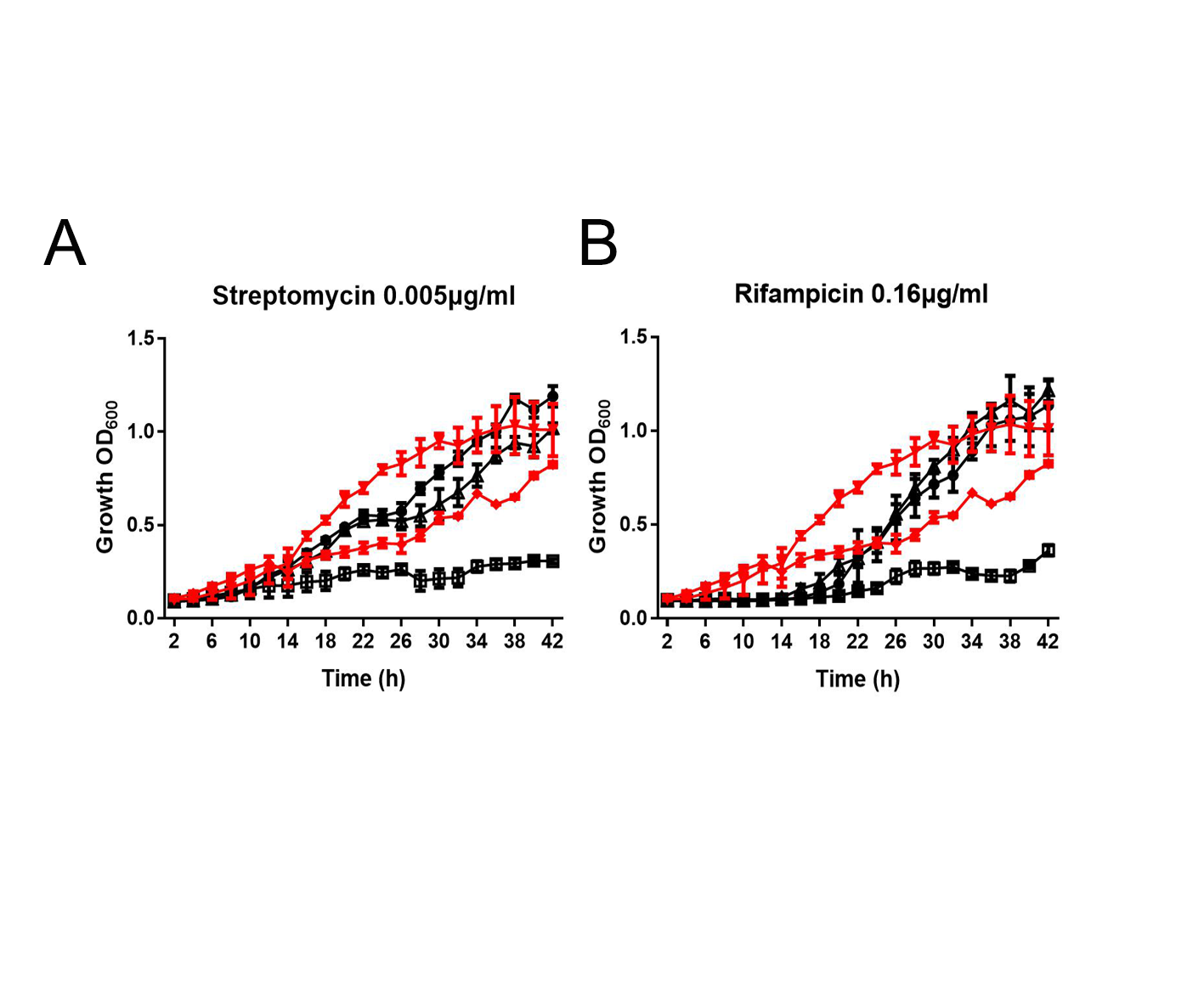

Supplement: FIGURE S2 — Deletion of greA altered susceptibility of antibiotics in M. smegmatis. WT, ΔgreA and comp-ΔgreA strains were exposed to 7H9 medium in the absent (control) or present of different antibiotics at 1/100th the MIC. The initial OD600 was 0.02. The growth curves were monitored for 42 h. (A) Streptomycin (MIC = 0.005 mg/L) and (B) Rifampicin (MIC = 0.16 mg/L). [file Image_2.TIF]

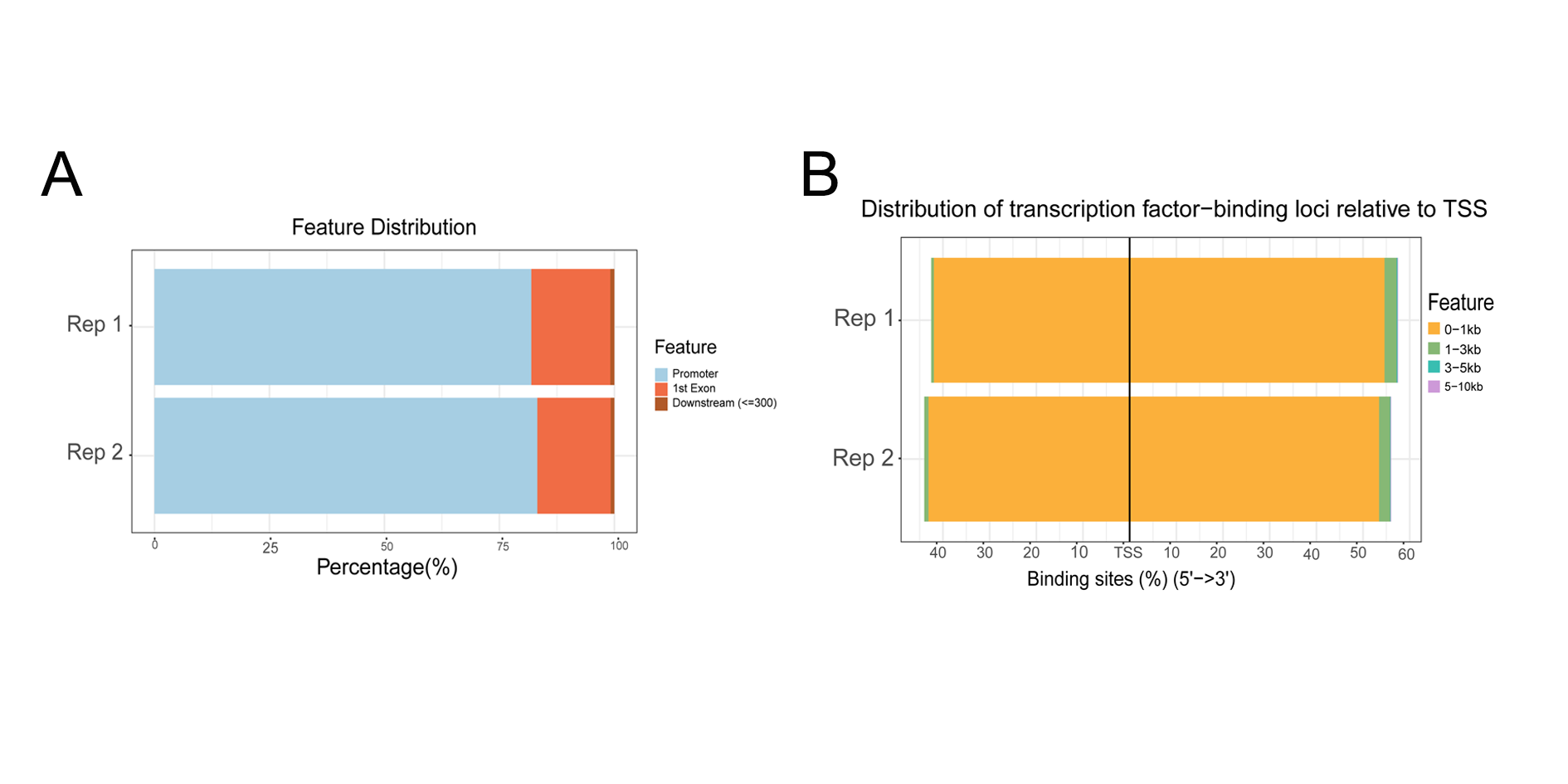

Supplement: FIGURE S3 — Analysis of binding sites identified from the ChIP-seq experiments. (A) Histogram showing the frequency distribution of distances between GreA ChIP-seq peak centers and TSSs. (B) Chromosomal binding map of HupB-FLAG. [file Image_3.tif]
